# Single femtosecond laser pulse–induced valence state conversion in BaFCl: Sm^3+^ nanocrystals for low-threshold optical storage

**DOI:** 10.1515/nanoph-2024-0181

**Published:** 2024-06-10

**Authors:** Biao Zheng, Lianzhong Deng, Jie Li, Yunhua Yao, Dalong Qi, Yuecheng Shen, Zhenrong Sun, Shian Zhang

**Affiliations:** State Key Laboratory of Precision Spectroscopy, School of Physics and Electronic Science, 12655 East China Normal University, Shanghai 200241, China; Fujian Key Laboratory of Functional Marine Sensing Materials, College of Material and Chemical Engineering, 12655 Minjiang University, Fuzhou 350108, China; Collaborative Innovation Center of Extreme Optics, Shanxi University, Taiyuan 030006, China; 12655 Joint Research Center of Light Manipulation Science and Photonic Integrated Chip of East China Normal University and Shandong Normal University, East China Normal University, Shanghai 200241, China

**Keywords:** rare-earth ion-doped nanocrystals, ion valence state conversion, optical storage, femtosecond laser field

## Abstract

Femtosecond laser-induced valence state conversion (VC) in solid materials has attracted significant research attention due to its potential application in ultra-high density optical storage, boasting advantages such as ultra-high recording speed, easy reading, and high signal-to-noise ratio. However, identifying appropriate materials and technological solutions conducive to efficient single-laser-shot recording remains a pivotal challenge for practical applications. In this work, we report single femtosecond laser pulse–induced VC in BaFCl: Sm^3+^ nanocrystals utilizing a 4F-configuration optical imaging system comprising two-dimensional scan galvo mirrors. For the first time, we experimentally reveal the luminescence mechanisms and channels of multiphoton absorption-induced Sm^2+^ ions under both single and multiple 800 nm fs laser pulses. Leveraging the highly efficient single femtosecond laser pulse induced VC, we demonstrate a prototype optical storage experiment by sweeping the recording laser pulse. Remarkably, a threshold pulse energy as low as ∼100 nJ for effective single-laser-shot recording in BaFCl: Sm^3+^ nanocrystals is obtained under the current experimental conditions. Our investigations offer profound insights into the physical mechanisms underlying femtosecond laser induced VC in solid materials, thereby promoting the prospects of VC based optical storage toward practical applications.

## 1 Introduction

The exponential surge in global data volumes demands innovative and cutting-edge data storage solutions [1], [2], [3]. Optical storage, which leverages laser-induced localized modifications in solid media such as crystal precipitation [4], [5], refractive index alteration [6], [7], ion valence conversion [8], [9], and nanostructure formation [10], [11], is emerging as a viable candidate for ultra-high-density, three-dimensional (3D) data storage. Particularly, optical storage based on laser-induced valence state conversion (VC) in rare-earth–doped solid materials [8] has garnered significant interest due to its ultrahigh recording speed, straightforward readability, and high signal-to-noise ratio. Researches were predominantly focused on luminescent materials doped with trivalent samarium (Sm^3+^) and europium (Eu^3+^) ions, which can provide profound and long-lasting electron trapping sites [12], [13]. Over the years, a number of works were conducted on VC in rare-earth–doped glass and crystals, such as phenomena and mechanism investigations with femtosecond laser pulses [14], [15], [16], [17], the influences of laser parameters, and other factors [18], [19]. Studies on VC in rare-earth–doped nanocrystals, like BaFCl: Sm^3+^, were also reported, like phenomena and mechanism investigations with nanosecond blue laser pulses [20] and UV-C lamp light [21], UV-C lamp light–induced VC for optical data storage [22], and grayscale fluorescence imaging [23]. For optical storage application, femtosecond laser–induced VC can provide rather high recording speed and is more favorable. Our latest findings revealed that photoreduction in BaFCl:Sm^3+^ nanocrystals can occur with rather high efficiency under excitation of 800 nm fs laser pulses [24]. However, the luminescence mechanisms and pathways of photoinduced Sm^2+^ ions under single and multiple femtosecond laser pulses could not be identified due to limitations of former experimental setup. Especially, single femtosecond laser pulse–induced VC is crucial for ultrahigh recording speed. Therefore, further identifying the underlying mechanisms and exploring the potential of this sample for single laser shot data recording are necessary. Recent studies have suggested the prospect of single femtosecond laser pulse–induced VC in rare-earth–doped glass for optical data storage [25], [26], with the data array recorded by moving the sample stage. Instead of controlling the sample stage, a simple and convenient method of scanning the laser pulse might be preferred.

In this work, we present the achievement of high-efficiency, low-threshold single laser shot recording in BaFCl:Sm^3+^ nanocrystals using an 800 nm fs laser pulse with a 4F-configuration optical imaging system incorporating two-dimensional (2D) scan galvo mirrors (GMs). We reveal the luminescence mechanisms of multiphoton-induced Sm^2+^ ions under both single and multiple femtosecond laser pulses. Meanwhile, we conduct a prototype optical storage experiment, which involves information recording with a single 800 nm fs laser pulse and reading with a 532 nm continuous-wave (cw) laser. Finally, we obtain the threshold for effective single laser shot recording by examining the relationship between the luminescence intensity of laser-induced Sm^2+^ ions and the femtosecond laser pulse energy.

## 2 Experimental designs

To obtain a homogeneous and transparent solution, 0.01 mol BaCl_2_·2H_2_O (99.9 %) and 0.0002 mol SmCl_3_·6H_2_O (99.9 %) were dissolved in a 25 mL aqueous solution and vigorously stirred at room temperature. Subsequently, 0.01 mol NH_4_F (98 %) was added to the mixture and stirred vigorously for 30 min. The resulting BaFCl:Sm^3+^ nanoparticles were precipitated by centrifugation and dried at 50 °C for 12 h. Further details involving the synthesis of BaFCl:Sm^3+^ nanoparticles can be found in our previous publication [24].

**Figure 1:**
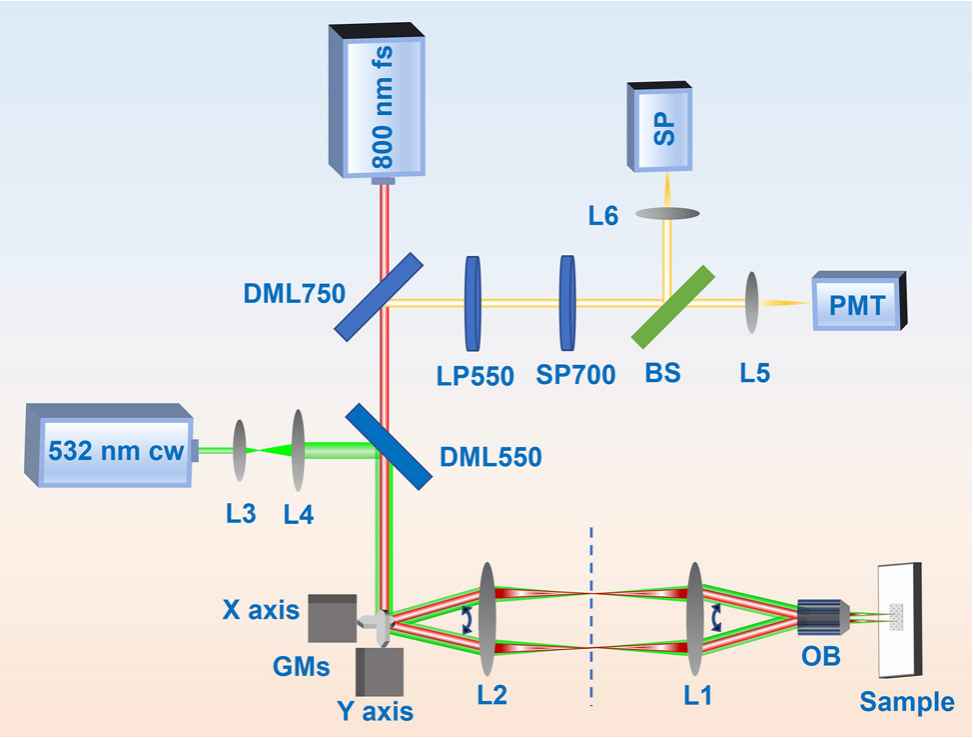
Schematic diagram of experimental setup. DML, long-wavelength pass dichroic mirror; GMs, 2D scan galvo mirrors; L1–L6, focusing lenses; OB, objective lens; LP, long-wavelength pass filter; SP700, short-wavelength pass filter; BS, beam splitter; PMT, photomultiplier tube; SP, spectrometer.

The experimental setup, as depicted in Figure 1, employs a femtosecond laser system as the primary excitation source. The system includes a titanium-sapphire mode-locked regenerative amplifier (Spitfire, Spectra Physics) that generates pulses with a central wavelength of 800 nm, a full-width at half-maximum (FWHM) bandwidth of approximately 20 nm, and a repetition frequency of 1 kHz. In our experiment, the 800 nm fs laser traverses through a 750 nm long-wavelength pass dichroic mirror (DML750), a 550 nm long-wavelength pass dichroic mirror (DML550), a 4F-configuration optical system comprising 2D scan GMs and two lenses (L1 and L2), and is focused onto the sample using an objective lens (OB) with a 0.25 numeric aperture (NA). The sample was prepared by uniformly coating a quartz plate with nanoparticles dissolved in cyclohexane. Subsequently, the fluorescence signal from the sample traverses through the OB, 4F optical system, GMs, DML550, DML750, and is then filtered by a 550 nm long-wavelength pass filter (LP550) and a 700 nm short-wavelength pass filter (SP700). This filtered fluorescence signal is split into two beams by a 50/50 beam splitter (BS), focused by lenses L5 and L6, and detected by a fiber spectrometer (SP, Ocean Optics MayaPro) and a photomultiplier tube (PMT), respectively. Furthermore, to read recorded information from divalent samarium ions induced by the 800 nm fs laser, a 532 nm cw laser is directed through a beam expander (L3 and L4), the DML550, GMs, 4F optical system, OB, and ultimately reaches the sample.

With the femtosecond laser operating at a repetition rate of 1 kHz, the time interval between adjacent pulses is 1 ms. Considering the fiber spectrometer’s integration time of no less than 7 ms, it is evident that independent luminescence detection under single femtosecond laser pulse excitation is not achievable. Therefore, in our experiment, single laser pulse irradiation on different sample points, with a spatial interval of approximately 20 μm, is facilitated by controlling the scan rate of the 2D GMs. The laser focal spot size is estimated to be around 2 μm using the conventional expression *d* = 4*fλ*/π*D*, where *f* represents the focal length of the OB, *λ* denotes the wavelength, and *D* signifies the diameter of the femtosecond laser beam. This configuration ensures that the luminescence signal detected by the time-integrated spectrometer solely originates from single femtosecond laser pulse irradiation. Conversely, for multiple femtosecond pulse excitation, the GMs remain stationary, and laser pulses continuously irradiate the same sample point.

## 3 Results and discussion

**Figure 2:**
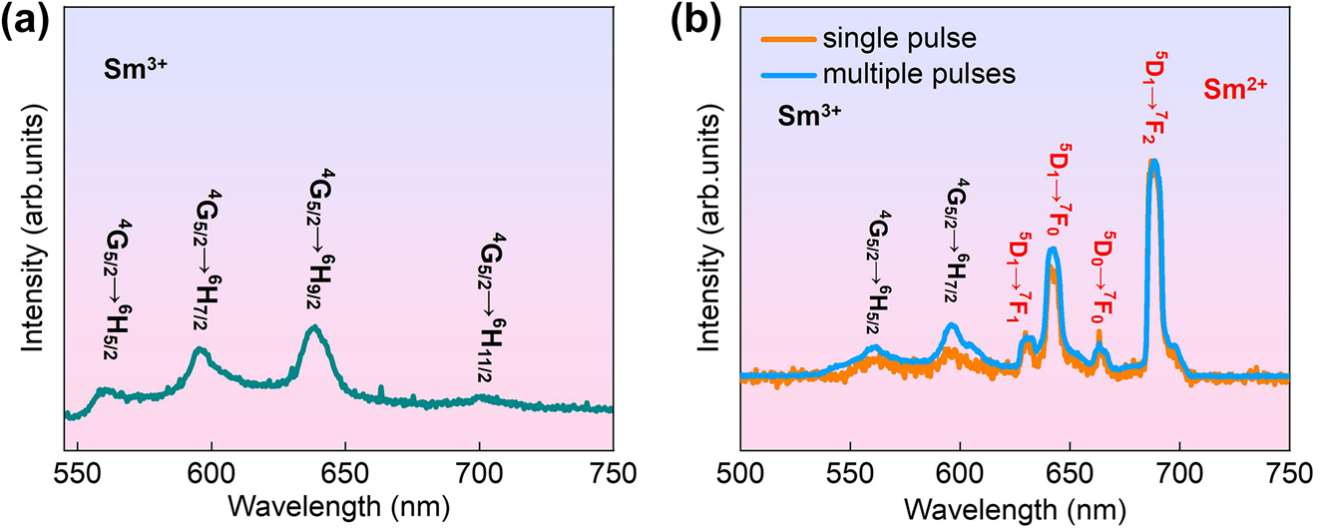
Luminescence spectra of the sample under excitation of 532 nm cw laser before femtosecond laser irradiation (a) and under excitation of single (orange curve) and multiple (blue curve) 800 nm fs laser pulses (b).

Before femtosecond laser irradiation, the sample is scanned using a 532 nm cw laser (∼20 mW) to verify the absence of detectable emission from Sm^2+^ ions. A typical spectrum of the scanned results is shown in Figure 2(a). The 532 nm laser excites the Sm^3+^ ion from the ground state ^6^H_5/2_ to the excited state ^4^F_3/2_, followed by nonradiative decay to the state ^4^G_5/2_. The luminescence peaks centered at approximately 560, 595, 639, and 700 nm correspond to the characteristic transitions of Sm^3+^: ^4^G_5/2_ → ^6^H_5/2_, ^6^H_7/2_, ^6^H_9/2_, and ^6^H_11/2_, respectively [27]. Subsequently, the luminescence signal from the sample under single femtosecond laser pulse irradiation (∼1.0 mW) is captured with the help of the scanning GMs. Figure 2(b) presents a typical spectrum (orange curve). The distinct new peaks emerging at approximately 630, 642, 660, and 688 nm are attributed to the transitions of Sm^2+^: ^5^D_1_ → ^7^F_0_, ^7^F_1_, ^7^F_2_, and ^5^D_0_ → ^7^F_0_, respectively [28]. It is important to note that the 700 nm short-wavelength pass filter, introduced to suppress the intense scattering signal from the irradiation laser pulses, cuts off other emission peaks at wavelengths exceeding 700 nm. The spectrum in Figure 2(b) clearly demonstrates that a single laser pulse can efficiently induce VC from Sm^3+^ to Sm^2+^ ions. Moreover, the emission spectrum of photoinduced Sm^2+^ ions under single femtosecond laser pulse irradiation provides solid experimental evidence that the multiphoton induced Sm^2+^ ions in BaFCl:Sm^3+^ nanocrystals under 800 nm fs laser pulse irradiation are initially prepared at highly excited ^5^D_*j*=0,1_ states and then decay to lower states, emitting observable luminescence. This phenomenon differs from previous reports on the systematic behavior in trivalent lanthanide charge transfer, which typically results in divalent ions directly at low-lying ^4^F_*j*=0–6_ states, particularly the ground state ^4^F_0_ [13]. For comparative analysis, the spectrum of the sample under fixed point irradiation with multiple femtosecond laser pulses is also recorded and displayed (blue curve) in Figure 2(b). The spectrum intensity with multiple femtosecond laser pulses is significantly stronger than that with a single femtosecond laser pulse. To better observe the relative heights of luminescence peaks, the two spectra are normalized at the peak at 688 nm. It is evident that the spectral peaks are similar in shape and position but exhibit slight differences in their relative intensities, particularly those of Sm^3+^ ions. These discrepancies can be partially attributed to the increasing local temperature of the sample point under multiple laser pulse irradiation.

**Figure 3:**
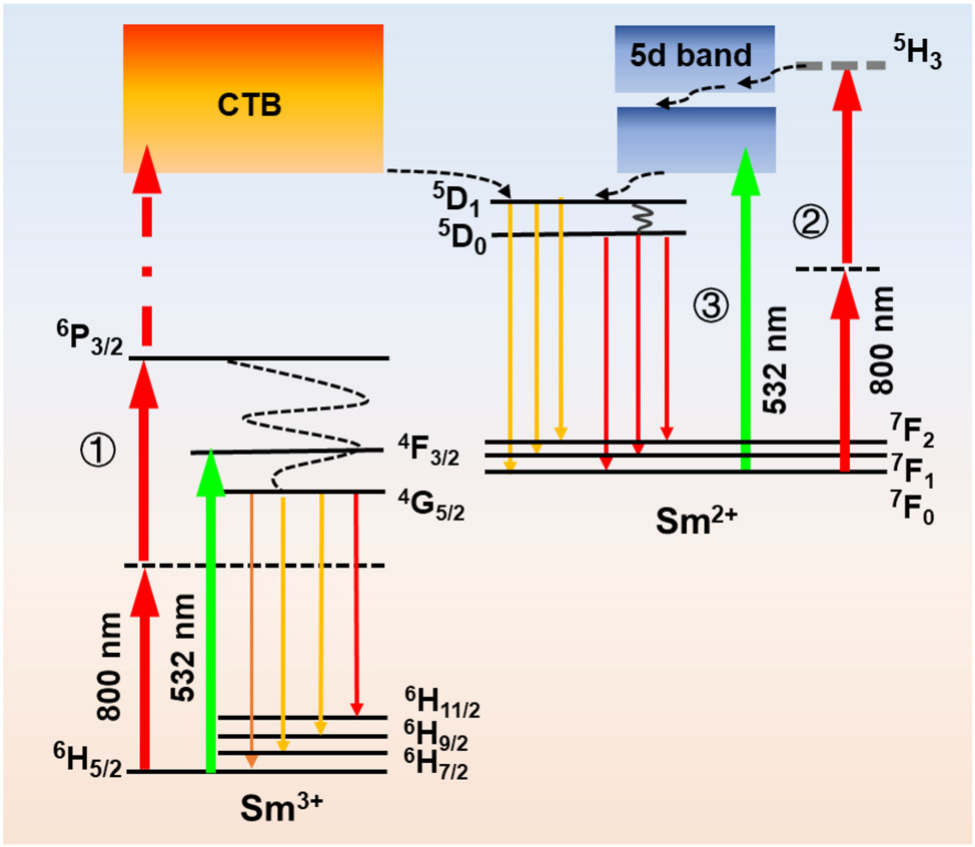
Energy level diagrams of samarium ions and possible transitions involved in the excitation, luminescence, and valence state conversion processes.

The energy level diagrams of samarium ions and the possible transitions are illustrated in Figure 3, involving the excitation, luminescence, and VC processes. Under femtosecond laser irradiation, the production and probe channels of Sm^2+^ ions are as follows: ① Resonance-mediated multiphoton absorption excites the Sm^3+^ ions from the ground state ^6^H_5/2_ to the charge transfer band (CTB) via the intermediate state ^6^P_3/2_. After capturing an electron from neighboring donors, the Sm^3+^ ion is reduced to a Sm^2+^ ion and enters the ^5^D_*j*=0,1_ states through nonradiative relaxation, followed by radiative decay to the low-lying ^4^F_*j*=0–2_ states, emitting luminescence at 630, 642, 660, and 688 nm, respectively. These emissions solely constitute the luminescence peaks of Sm^2+^ ions observed in the spectrum under single femtosecond laser pulse irradiation. Under multiple femtosecond laser pulses, the contribution of channel ① weakens, and another channel emerges, playing a predominant role. ② The former laser pulse–induced Sm^2+^ ions can be excited from the ground state ^7^F_0_ to highly excited 4f states with a mean energy of approximately 25,000 cm^−1^, denoted as ^5^H_3_ for simplicity, by simultaneously absorbing two photons from subsequent laser pulses, akin to the case of two-photon absorption in Eu^2+^ doped materials [29]. Since one-photon absorption at 800 nm does not meet the energy requirement to excite the luminescence of Sm^2+^ at 688 nm, two-photon absorption at 800 nm is needed. For two-photon absorption, the transition from the 4f ground state to the 5d band is parity forbidden and strongly suppressed, while the transitions within 4f multiplet are parity allowed and more preferred. Interconfigurational relaxation can transfer the ions in state ^5^H_3_ to the overlapping 5d band, followed by nonradiative relaxation to the lower ^5^D_*j*=0,1_ states. Upon radiative decay to the low-lying ^4^F_*j*=0–2_ states, luminescence at 630, 642, 660, and 688 nm can be observed. It is noteworthy that the ^5^D_2_ state of the Sm^2+^ ion lies within the tail of the 5d band [30], and emission is not observed here. Channel ③ corresponds to probing photoinduced Sm^2+^ ions with a 532 nm cw laser, as described in the following optical information readout section.

**Figure 4:**
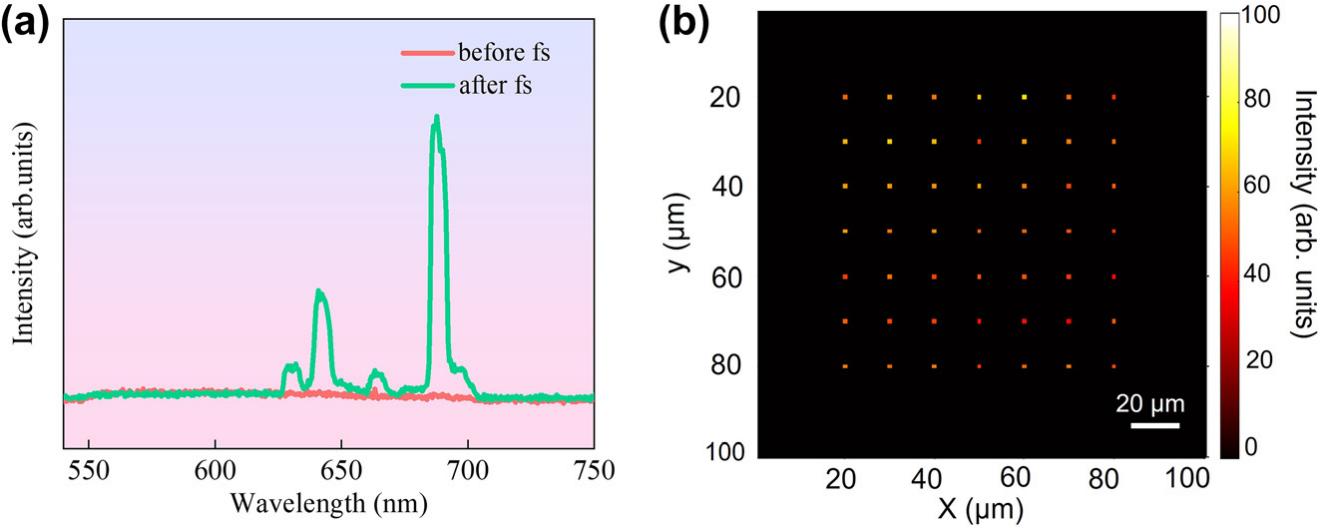
2D optical information “writing” and “reading”: (a) spectra before and after femtosecond laser “writing.” (b) 2D images of the information array “reading” by 532 nm cw laser.

As a prototype experiment, 2D optical information “writing” with a single 800 nm fs laser pulse and “reading” with a 532 nm cw laser is conducted using the optical imaging system comprising the 2D scan GMs. Figure 4(a) displays the representative luminescence spectra of the sample points under 532 nm cw laser excitation before (red curve) and after (green curve) single femtosecond laser pulse “writing.” Since a high-power 532 nm laser beam can “erase” the Sm^2+^ ions back to Sm^3+^ ions [8], the power of the “reading” laser beam is intentionally reduced to approximately 3.0 mW. Before information “writing,” no discernible spectral peaks from either Sm^3+^ or Sm^2+^ ions are observed (red curve). After information “writing,” strong luminescence peaks centered at 632, 642, 660, and 688 nm, exclusively from laser pulse-induced Sm^2+^ ions, are detected. Figure 4(b) presents a 2D mapping image of the central portion of the information array under 532 nm cw laser “reading” with optical signal detected by the PMT. The signal from the PMT is continuously collected by the computer during laser scan. And the photon signal at fixed time interval after proper background subtraction is accumulated and taken as an image point of the micro photo. The average image contrast of the data points is approximately 50:1. The irradiating femtosecond laser pulses have an average power of approximately 1.0 mW with a repetition rate of 1 kHz, corresponding to a single pulse energy of approximately 1.0 μJ. The high signal-to-noise ratio and reliable information recording depicted in Figure 4 demonstrate that VC from Sm^3+^ to Sm^2+^ ions can occur with high efficiency under single femtosecond pulse irradiation. Clearly, a femtosecond laser pulse with even lower energy can also be utilized for effective information “writing” while maintaining a reasonable signal-to-noise ratio.

The different colors of the data points in the micro photo of Figure 4(b) can be ascribed to a few reasons, such as the pulse-to-pulse fluctuation in energy of the femtosecond laser for “writing,” the fluctuation in absorption and luminescence of nanocrystals due to their nonuniformity in size, morphology, and surface condition, the slight change of the focusing spot during scanning and the misalignment of the optical system. In practical application of data storage, the influence of this deviation can be ignored so long as the intensity of the luminescence signal is well above the background noise. Surely, this deviation can be greatly suppressed by optimizing the aforementioned factors.

Besides, a high-power probe laser irradiation can “erase” the Sm^2+^ ions back to Sm^3+^ ions. The kind of bleaching behavior has strong dependences on the irradiation laser power and time, as reported in our latest research [24]. For this reason, the power of the 532 nm cw probe laser is intentionally reduced to approximately 3.0 mW to minimize the bleaching effect. Particularly, the usage of the 4F-configuration 2D laser scan system greatly shortens the irradiation time of the probe laser and helps suppress the bleaching effect further. In the experiment there is no observable decrease in the average image contrast of the data points after a few cycles of data readout. In fact, considering the strong signal-to-noise ratio shown in Figure 4, the probe laser power can be further greatly reduced.

**Figure 5:**
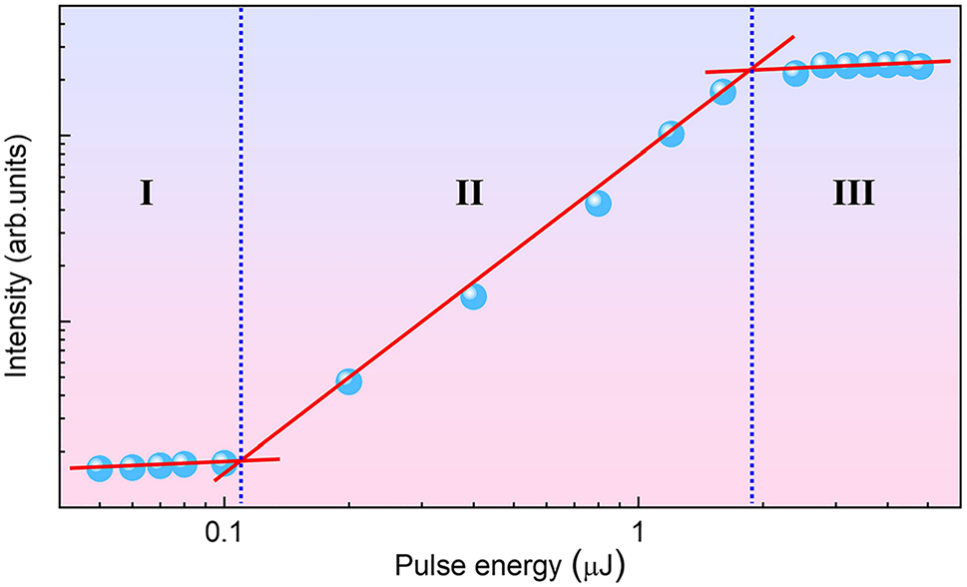
Dependence of the detected luminescence signal intensity from photoinduced Sm^2+^ ions (solid circles) on the femtosecond laser pulse energy (log–log plot).

To investigate the single laser shot threshold for optical information recording, the energy of the “writing” femtosecond laser pulse is varied from 0.05 to 5.0 μJ, while the “reading” 532 nm cw laser remains at 3.0 mW. Figure 5 illustrates the dependence of the detected luminescence signal from photoinduced Sm^2+^ ions on the laser pulse energy in a log–log plot (solid circles). According to the piecewise fittings (solid lines), when the laser pulse energy ranges from 0.05 to 0.1 μJ, there is no discernible increase in the luminescence signal intensity (labeled as I zone). However, if the laser pulse energy is adjusted from 0.1 to 2.0 μJ, a significant increase in the signal intensity is observed, indicating the threshold of laser-induced VC is as low as 0.1 μJ (100 nJ) (labeled as II zone). Once the laser pulse energy exceeds 2.0 μJ, the signal intensity gradually stabilizes (labeled as III zone).

Table 1 summarizes the energy thresholds of single femtosecond laser pulse for VC-based optical storage application reported so far. The sample material, NA of objective lens, recording laser, probe wavelength, and the corresponding threshold are listed. As an initial information recording demonstration, an objective lens with 0.25 NA is utilized in our current experiment. By employing an objective lens with a higher NA, the energy of the single laser pulse required for “writing” can be significantly reduced. For instance, the laser pulse energy can reduce tenfold by using an objective lens with 0.8 NA. This implies that a single laser pulse of tens of nJ could provide the same level of information recording. Naturally, a higher detection sensitivity for “reading” would be required accordingly, as the luminescing area diminishes.

**Table 1:** Summary of the energy thresholds of single femtosecond laser pulse for VC-based optical storage application.

Samples	N.A.	Recording laser	Probe wavelength	Thresholds	References
Sm^3+^-doped glass^a^	0.80	680 nm	1,030 nm	250 nJ	[25]
Eu^3+^-doped glass^b^	0.80	435 nm	1,030 nm	60 nJ^c^	[26]
BaFCl:Sm^3+^ nanocrystal	0.25	688 nm	800 nm	100 nJ	This work

^a,b^Sodium aluminoborate glass; ^c^with UV-light preprocessing.

For single femtosecond laser pulse–based optical storage, the data recording speed can be ultrahigh and is basically limited by the laser pulse repetition rate and the tilting speed of the scanning galvo mirrors or the moving speed of the sample stage. In practical application, it is simpler and more convenient to control the tilt of a silver mirror than the motion of a sample stage. Besides, the scanning area on the sample can be conveniently scaled by selecting an appropriate focus length ratio of the 4F lens (L1, L2) to the objective lens (OB). To realize three-dimensional data storage, the BaFCl: Sm^3+^ nanocrystals can be embedded into transparent glass [20] or polymer materials of similar refractive index for less scattering. Simply moving the sample along the direction of the recording laser beam axis, data information can be conveniently recorded in different layers of the media with the help of the 4F-configuration 2D scan optical system.

## 4 Conclusions

In summary, we have demonstrated single laser shot optical storage based on the single femtosecond laser pulse–induced VC in BaFCl: Sm^3+^ nanocrystals. Using a 4F-configuration optical imaging system comprising 2D scan galvo mirrors, we investigate the luminescence mechanisms of the photoinduced Sm^2+^ ions under both single and multiple 800 nm fs laser pulses. Experimental evidence clearly indicates that the femtosecond laser–induced Sm^2+^ ions are initially prepared at highly excited 4f electronic states of ^5^D_0,1_, followed by radiation decay to low-lying ^4^F_*j*=0–2_ states. This novel phenomenon differs from previous reports on the systematic behavior in trivalent lanthanide charge transfer. Highly efficient and reliable 2D optical recording has been conducted with high signal-to-noise ratio. The dependence of luminescence signal from the photoinduced Sm^2+^ ions on the femtosecond laser pulse energy has revealed a single laser shot threshold for effective information recording as low as ∼100 nJ. Compared to a mechanically mobile sample stage, the 4F-configuration laser scan system can bring quicker motion and a broader scan range, offering higher recording efficiency and greater convenience in optical storage applications. Our studies shed light on the mechanisms of femtosecond laser–induced VC in rare-earth ion-doped luminescent materials and represent a forward step toward optical storage applications demanding high recording efficiency, strong signal-to-noise ratio, and low laser pulse energy threshold.
